# Heat-Related Illnesses Transported by United States Emergency Medical Services

**DOI:** 10.3390/medicina56100543

**Published:** 2020-10-17

**Authors:** Susan Yeargin, Rebecca Hirschhorn, Andrew Grundstein

**Affiliations:** 1 Department of Exercise Science, University of South Carolina, Columbia, SC 29208, USA; 2 School of Kinesiology, Auburn University, Auburn, AL 36849, USA; rmh0075@auburn.edu; 3 Department of Geography, University of Georgia, Athens, GA 30602, USA; andrewg@uga.edu

**Keywords:** heat stroke, heat exhaustion, environmental conditions

## Abstract

*Background and objectives:* Heat-related illness (HRI) can have significant morbidity and mortality consequences. Research has predominately focused on HRI in the emergency department, yet health care leading up to hospital arrival can impact patient outcomes. Therefore, the purpose of this study was to describe HRI in the prehospital setting. *Materials and Methods:* A descriptive epidemiological design was utilized using data from the National Emergency Medical Services (EMS) Information System for the 2017–2018 calendar years. Variables of interest in this study were: patient demographics (age, gender, race), US census division, urbanicity, dispatch timestamp, incident disposition, primary provider impression, and regional temperatures. *Results:* There were 34,814 HRIs reported. The majority of patients were white (*n* = 10,878, 55.6%), males (*n* = 21,818, 62.7%), and in the 25 to 64 age group (*n* = 18,489, 53.1%). Most HRIs occurred in the South Atlantic US census division (*n* = 11,732, 33.7%), during the summer (*n* = 23,873, 68.6%), and in urban areas (*n* = 27,541, 83.5%). The hottest regions were East South Central, West South Central, and South Atlantic, with peak summer temperatures in excess of 30.0 °C. In the spring and summer, most regions had near normal temperatures within 0.5 °C of the long-term mean. EMS dispatch was called for an HRI predominately between the hours of 11:00 a.m.–6:59 p.m. (*n* = 26,344, 75.7%), with the majority (27,601, 79.3%) of HRIs considered heat exhaustion and requiring the patient to be treated and transported (*n* = 24,531, 70.5%). *Conclusions:* All age groups experienced HRI but particularly those 25 to 64 years old. Targeted education to increase public awareness of HRI in this age group may be needed. Region temperature most likely explains why certain divisions of the US have higher HRI frequency. Afternoons in the summer are when EMS agencies should be prepared for HRI activations. EMS units in high HRI frequency US divisions may need to carry additional treatment interventions for all HRI types.

## 1. Introduction

The term “heat-related illness” (HRI) encompasses many different conditions as it includes any ailment that a health care provider perceives as being related to warm environmental temperatures. The United State (US) experiences warm to hot environmental conditions throughout the year depending on the region of the country. With climate warming across the world, HRI is a public health concern [1]. Heat-related illness can include commonly diagnosed conditions, such as heat cramps, heat exhaustion, heat syncope, and heat stroke. However, ICD-10 codes also allow for HRI to be ambiguously coded, such as “heat fatigue” and “effects of heat and light”. Heat cramps, more commonly known by sports medicine organizations as exercise-associated muscle cramps, are involuntary contractions of skeletal muscles [2,3,4]. Heat exhaustion is defined as collapse due to cardiovascular insufficiency in which dehydration and/or sodium losses are the typical etiologies [2,3,4]. Heat syncope typically occurs in populations that have been standing for long periods of time (i.e., soldiers, marching band members) or individuals who have suddenly stopped at a finish line. Blood pools in the lower extremities and the patient faints as a protective mechanism to return normal blood flow to the heart and brain [2,3,4]. Heat exhaustion, heat syncope, heat cramps, and dehydration compose the majority of HRI evaluated in emergency departments (ED) [5,6,7,8,9].

Heat stroke, on the other hand, is a deadly HRI as the individual’s core body temperature can rise dangerously above 40 °C (>104.5 °F) [10]. This results in significant central nervous system dysfunction and organ failure [10,11,12]. Etiologies can include environmental heat gain and/or exertional metabolic heat generation. Treatment requires aggressive body cooling and monitoring of vital organs [3,4,10,13]. The Center for Disease Control (CDC) estimated that a total of 3442 deaths were heat-related between 1999 and 2003 (annual mean: 688) [14]. The CDC report indicated that the number of heat-related deaths is actually underestimated.

The vast majority of HRI research has been conducted in EDs, at the state or research network level [5,6,7,8,9,15]. HRIs differ significantly in terms of diagnoses, treatment interventions, and case disposition, therefore making descriptive research during the different stages of HRI (i.e., incident, prehospital care, emergency department, hospitalization, and post-hospitalization) imperative to provide a foundation for future clinical research. Only a few studies have been conducted in the prehospital setting, focused on organized sport populations or the military [16,17,18], leaving understanding of HRI in the general population unexamined. Understanding HRI in this setting is vital as transportation from the incident to the emergency department can play a large role in health outcomes [19,20]. Mortality rates can be reduced by more than 20% when cooling is completed within 60 min, emphasizing the need for prompt prehospital recognition by emergency medical services (EMS) and fast transportation to the nearest facility [13]. To date, there has been no HRI research conducted using data from EMS units. The National EMS Information System (NEMSIS), a standardized EMS database, presents an opportunity to examine HRI from the perspective of EMS across the US. Therefore, the purpose of this study was to describe EMS activations for HRI using the NEMSIS.

## 2. Materials and Methods

### 2.1. Study Design

A descriptive epidemiological design was conducted utilizing data from the NEMSIS for the 2017–2018 calendar years. These years were chosen because the NEMSIS began using ICD-10-CM codes in 2017. Emergency medical services activations included in this study were limited to 9-1-1 responses for individuals aged infancy (0 years) to 100 years of age in which an HRI was documented. The current study was conducted as part of a larger examination of injuries presenting to EMS.

### 2.2. Study Procedures

A data request was submitted to the NEMSIS for the 2017 and 2018 Public-Release Datasets. The NEMSIS collects data voluntarily reported by participating EMS agencies across the US using compatible documentation software. The NEMSIS includes data from both publicly and privately funded EMS agencies in the US. EMS personnel provide services at three main certification levels (emergency medical technician, advanced emergency medical technician, and paramedic), each requiring completion of a standardized curriculum of didactic with hands-on training and continuing education to maintain certification. EMS within the US are available for every individual; however, response time and the level of responding providers (basic life support or advanced life support) vary depending on the urbanicity or ruralness of the area and type of EMS available. EMS agencies bill for services provided, depending on the individuals’ insurance, and costs vary for how much the patient must pay out of pocket, if any. EMS personnel provide care under the direction of their agency’s EMS medical director and must follow their agency’s approved protocols. EMS protocols vary by state and EMS agency.

For each EMS activation, a patient care report is completed by the EMS provider. Emergency medical services agencies determine which elements to include in their documentation but must include required national and state-level elements. The 2017 Public-Release Research Dataset included over 7.9 million EMS activations from 4016 EMS agencies across 35 states and territories [21]. The 2018 Public-Release Research Dataset included over 22.5 million EMS activations from 9599 EMS agencies across 43 states and territories [21]. Each public release dataset contained all required national-level variables. Not all variables are mandated to be completed by the EMS provider in the patient care report and some allow the EMS provider to select “Not Applicable”, resulting in varying completeness of exported reports. Data received from participating agencies were checked by the NEMSIS Technical Assistance Center for completeness, logical consistency and formatting, and quality assurance. All data were de-identified.

A heat-related illness was operationally defined as an EMS activation within the NEMSIS based on ICD-10-CM codes of the T.67 series (as selected within the patient care report and determined by the EMS provider). Codes within this series were categorized as either (1) heat stroke, (2) heat syncope, (3) heat cramp, (4) heat exhaustion, or (5) other. All ICD-10-CM codes within this series and their corresponding heat-related illness category are presented in a supplemental figure.

### 2.3. Variables of Interest

The NEMSIS variables of interest in this study were: patient demographics (patient age, patient gender, patient race), US census division, urbanicity, dispatch timestamp, incident disposition, and primary provider impression. Patient age (in years), US census division [22], and urbanicity [23] were calculated and provided by the NEMSIS but not directly entered by the EMS provider completing the patient care report. Dispatch time stamp included the month, day, and time in which EMS was activated. Incident/patient disposition was the resulting treatment and/or transport of the EMS event (e.g., call cancelled prior to arrival at scene, patient refused evaluation/care, patient treated and transported by EMS). Provider’s primary impression was the EMS provider’s impression of the patient’s predominant problem or most significant condition which led to the management procedures (treatment, medication, or monitoring).

### 2.4. Census Region Temperature Patterns

Regional temperature patterns were identified using the climate division dataset [24]. The monthly average maximum temperature for each state within the contiguous US was computed for the study period (January 2017 to December 2018). These data were compiled into seasons as winter (December, January, February), spring (April to May), summer (June to August), and fall (September to October), and then aggregated by census region using area-weighted averages. A similar approach was used using the long-term (1981–2010) mean values. The differences between the study period mean maximum and the long-term mean maximum by season were compared to determine if the study years were hotter or cooler than average.

### 2.5. Statistical Analysis

Data were analyzed using SAS^®^ software (Version 9.4, SAS Institute Inc., Cary, NC, USA). Frequencies were calculated for all variables. Means and standard deviation were calculated for patient age. Age groups were created for provider impression based on life stages (children <13, secondary school 13–18, post-secondary school 19–24, adult 25–64, and elderly >64). Cases in which a required variable was coded as “not applicable” or “not recorded” by the NEMSIS were combined as “not documented” for descriptive reporting. In the data set provided by NEMSIS, cells that provided no information were considered “missing”.

## 3. Results

### 3.1. Patient Demographics

**Figure 1:**
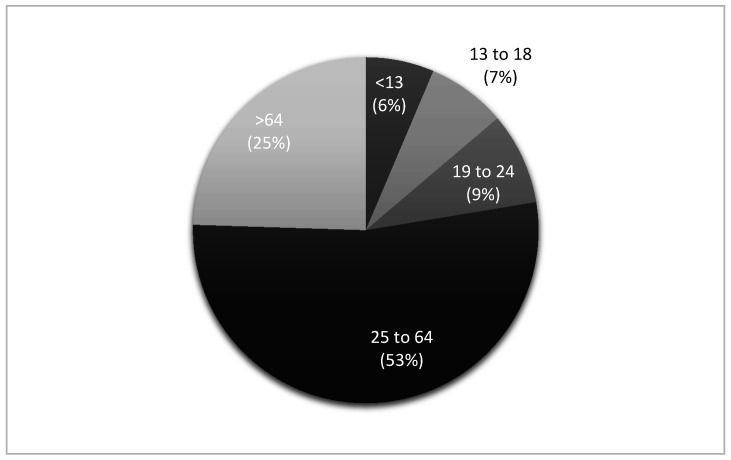
Frequency of age group that experienced a heat-related illness. Missing = 405.

There were 34,814 HRIs reported in NEMSIS for the years 2017 and 2018. The average age of an individual that required EMS activation for an HRI was 46.4 ± 22.9 years, with the majority (*n* = 18,489, 53.1%) of patients in the 25 to 64 age group. Frequency of age groups that experienced an HRI are presented in Figure 1. White people (*n* = 10,878, 55.6%) and males (*n* = 21,818, 62.7%) composed the majority of patients. Gender was not documented in 247 cases (0.7%). A breakdown of HRI frequency by race is provided in Table 1.

### 3.2. US Census Division and Urbanicity

The most (*n* = 11,732, 33.7%) HRIs occurred in the South Atlantic division of the United States. The remaining divisions composing the southern region of the US (East South Central and West South Central) also composed a large portion (*n* = 5922, 17.0%) of HRI EMS activations. A breakdown of HRI frequency in each census division is provided in Table 2. Urban areas experienced the majority (*n* = 27,541, 83.5%) of HRI. Suburban areas only constituted 5.8% of HRIs (*n* = 1916), whereas rural and wilderness areas composed the remaining 10.7% (*n* = 3538).

### 3.3. Dispatch Date and Time

**Figure 2:**
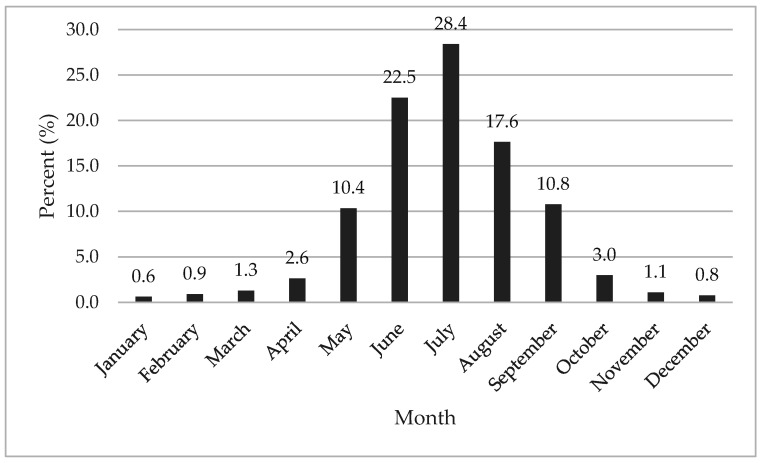
Heat-related illness by month.

Most (*n* = 23,873, 68.6%) EMS activations for HRI occurred between the summer months (June–August). Frequency of HRI by each month is provided in Figure 2. The beginning (1st–15th) and end (16th–31st) of the month were evenly divided (*n* = 17,437, 50.1%; *n* = 17,377, 49.9%, respectively) for HRI EMS activations. EMS dispatch was called for an HRI between the hours of 11:00 a.m.–2:59 p.m. (*n* = 13,282, 38.2%) and 3:00 p.m.–6:59 p.m. (*n* = 13,062, 37.5%) the most. The remaining frequencies in which EMS dispatch was called were between 7:00 pm and 12:59 a.m. (*n* = 4400, 12.6%), 1:00 a.m. and 6:59 a.m. (*n* = 637, 1.8%), and 7:00 a.m. and 10:59 (*n* = 3433, 9.9%).

### 3.4. Census Division Temperature Patterns

All regions had similar seasonal patterns, with the coldest temperatures in winter, intermediate temperatures in spring and fall, and the hottest temperatures in the summer (Table 3). Yet, there were diverse seasonal maximum temperatures among regions. Throughout the year, the hottest divisions were East South Central, West South Central, and South Atlantic, with the warmest winter temperatures (13.5–15.8 °C) and peak summer temperatures in excess of 30.0 °C. In contrast, the East North Central, West North Central, and New England regions had the coldest winter temperatures (0.1–2.1 °C) and the mildest summer temperatures (25.0–28.8 °C). The remaining divisions had intermediate temperatures.

The degree of difference in maximum temperatures from the long-term mean varied by division and season. Most notably, winter temperatures were above average across the country, ranging from 0.3–1.9 °C. In the spring and summer, many divisions had near normal temperatures within 0.5 °C of the long-term mean but the Mountain and West South Central divisions were over 1 °C warmer in the spring and the Pacific and Mountain divisions were over 1 °C warmer in summer. In the fall, most divisions had near-normal conditions within 0.5 °C of the long-term mean.

### 3.5. Provider Impression and Incident Disposition

The majority (27,601, 79.3%) of HRIs were considered heat exhaustion by EMS providers. Heat stroke also comprised a large portion (*n* = 5955, 17.1%) of HRI. Heat syncope (*n* = 298, 0.9%), heat cramps (304, 0.9%), and other (*n* = 656, 1.9%) composed small percentages of the remaining cases. Provider impression frequencies by age group are provided in Table 4. Heat exhaustion composed the most HRI in each age group. Heat stroke occurred the most frequently (*n* = 2890, 8.3%) in the 25 to 64 year old age group. The vast majority (*n* = 24,531, 70.5%) of HRIs required the patient to be treated and transported (Table 5).

## 4. Discussion

### 4.1. Patient Demographics

The purpose of our study was to describe EMS activations for HRI using the NEMSIS. The most common clinical scenario for HRI based on our results is a white male between the age of 24 and 65. The mean age for the HRI patient was 46. This was unanticipated as most HRI research has identified young children and the elderly in classic heat stroke cases and high school or college athletes in exertional heat stroke cases [10,17,18,25]. Our study brings to light a new age group (25–64) that may be at risk for HRI. This age group may be at risk for HRI for a variety of reasons including: physical activity engagement, occupational exposure, increased body mass index, and comorbidities [10,11]. It could be that health care entities may need to develop public education regarding heat illnesses for this age group. Occupational health surveillance can also help identify possible HRI prevention strategies in this age group [26]. It is valuable to point out that no age group was immune from HRI. The over 5500 cases between the 13- to 24-year-old age group emphasizes a need to examine exertional heat illness related to sport and physical activity in an effort to reduce risk in middle school to high school students [17,18]. Young children, such as those in the <13 year old age group, with HRI have the highest adjusted odds ratio of death in the ED compared to other age groups [6]. “Natural/environment” heat-related deaths have been in the top 10 causes of unintentional injury fatalities in children since 2010 [14]. The dataset does not provide a detailed explanation of settings which may provide more insight into HRI prevalence in different age groups.

Heat related illnesses are considered multifactorial by experts, nonetheless correlates that are associated with hospitalization and fatalities have been identified in adult and pediatric populations [3,4,5,6,7,15,19,20,27,28,29,30]. Our data reported that HRI happened most often in white people. Research regarding race and HRI is inconclusive [30]. Various research has reported higher incidence of HRI in black people. Genetic differences in heat tolerance have not been demonstrated in previous research. Author opinion suggested racial differences are probably attributed to socioeconomic status, physical health, urbanicity, and occupational exposure more so than physiological differences [30]. Our additional variables of interest do not provide insight as to why white people constituted the majority of cases in the dataset.

Males compose the majority of ED and EMS encounters [31,32,33], so it was no surprise they were the majority of HRI patients. A meta-analysis of HRI incidence reported males have higher rates than females across deaths, hospital admission, lifespan, and severity. Yet, the authors could not develop a conclusion on consistent risk factors to explain why [34]. Our dataset does not provide information regarding events leading up to the HRI event, which could help future research understand why males have a higher prevalence of HRI. Particularly, information on the occupation of those with HRI in the adult population may shed light on the effects of occupational health in heat waves [35]. Females still constituted 37.3% of HRI in our study. Research from the military has shown females have an increased adjusted odds of heat illness [36] but this could be exclusive to exertional heat illnesses, whereas our study was more inclusive of all types of HRI.

### 4.2. US Census Division and Urbanicity

Most HRIs occurred in the South Atlantic and other census divisions within the lower half of the contingent US. This aligns with research examining exertional heat illnesses that have noted that southern states and regions experience greater HRI incidence [5,17,37]. These divisions experience greater environmental temperatures, in which numerous studies have indicated that 27.7 °C is a critical threshold at which the incidence of HRI increases [7,38,39,40,41]. This threshold is commonly reached in the south, not just in summer but the spring and fall as well. However, both ED research and case series have reported that HRI occurs in every division within the United States [5,14,25,37].

Urban areas reported the most HRIs in which EMS was activated [30]. A broad body of research has documented higher air temperatures within urban compared with rural areas (i.e., the “urban heat island effect”) [42]. This difference in temperature is associated with multiple factors, including urban geometry, the thermal properties of building materials, lack of vegetation, and heat released from human activities [42]. We should acknowledge that these results could also be a factor of those EMS agencies that participate in the NEMSIS. Previous descriptions of this database indicate most (53%) agencies reside in an urban area [43]. Lastly, urban areas are more populated than rural locales. High HRI frequency in urban areas is most likely a result of more people living in those spaces.

### 4.3. Dispatch Date and Time

Summer months comprised the greatest amount of HRI EMS activations. This has been introduced and confirmed in previous HRI research within ED and sport settings [5,6,7,8,9,17]. The summer months coincide with individuals being outdoors with traditional recreation activities and hotter temperatures throughout the contingent US. The day within the month did not seem to impact the frequency of EMS activations for HRI. On the other hand, time of day did seem to influence frequency of HRI. Most EMS activations occurred between 11:00 a.m. and 6:59 p.m. This can be explained by not only the ambient air temperatures significantly contributing to convective heat gain but also the sun contributing to short and long wave solar radiation heat gain [44]. The sun is highest in the sky during this time frame as the average time of sunset in the summer is between 7 and 9 p.m. EMS should be prepared for HRI during the summer months, particularly from 11 a.m. to 7 p.m.

### 4.4. Census Division Temperature Patterns

The seasonal pattern of heat-related illness with a peak in the summer is consistent with the hottest period of time in the US. Indeed, this is supported by previous ED HRI research that has noted a threshold of 27.7 °C, which was reached, on average, in six of the nine divisions in the summer and is close to the average maximum temperature in a seventh region (27.1 °C). Compared to the long-term mean, regional maximum summer temperatures during the study time frame would be considered near-normal for all divisions but the Mountain and Pacific. This is relevant because past research indicates that the absolute temperature reached is not necessarily the key risk factor in heat stroke. Rather, in milder climates, such as in northern divisions of the US, it is hotter than normal temperatures that are a detrimental risk factor in fatal heat stroke cases [37]. We observed geographic variability in HRIs, yet the hottest three divisions accounted for 50% of the HRIs. Future research should encourage EDs to record the environmental conditions in the patient history. Ultimately, our data support that as temperatures get warmer in the summer, HRI frequency increases in each division. With forecasts of hotter conditions and more frequent heat waves, EMS units should be prepared for increased HRIs.

### 4.5. Provider Impression and Patient Disposition

Heat exhaustion was the preponderance of provider impressions. Heat exhaustion is generally defined as the inability to continue activity in the heat because of cardiovascular insufficiency [2,3,4]. Since hypohydration is considered the primary etiology of heat exhaustion, EMS agencies should be prepared, particularly in the summer months, with oral or intravenous fluids for treatment.

Heat stroke also had a clinically meaningful frequency of HRI cases. Heat stroke, both exertional and classic, can result in significant morbidity or mortality [10,11]. Since the two types of heat stroke occur in different populations and have different etiologies, differentiation in future research is important. Additionally, it is imperative for EMS responders to understand both types for faster recognition. In a retrospective cohort study of heat stroke patients, 69% of cases were not correctly diagnosed in the prehospital setting [45]. Patients who had treatment delays because of misdiagnosis had higher rates of multi-organ failure and longer hospitalizations [45]. Our proportion of heat stroke cases and previous misdiagnosis research underline the importance of the EMS provider’s evaluation.

A small percentage of HRI cases in our data set were categorized as “other”. The ICD-10-CM codes within this category are relatively ambiguous. In the ED, 15% to 66% of HRI cases are not given a specific diagnosis [6,8,9,46]. A thorough evaluation (i.e., history, core body temperature) can typically produce a diagnosis within the most common HRI types. A more definitive diagnosis can benefit EMS providers in order to determine targeted transport management [47].

To our knowledge, we are the first to provide HRI type by age group in which children are involved in the dataset. This is important as the majority of HRI research is focused on adolescent and adult populations. Very few have examined pediatric HRI, and those who have, report patient outcomes in aggregate with adults [6,8,9]. Children (<18 years old) in our dataset experienced each type of HRI, with heat exhaustion and heat stroke composing the majority of HRI in this age group. Even though children have efficient thermoregulatory systems [48], they may lack the decision-making ability or physical ability to remove themselves from situations in which an HRI may occur. More research and public awareness is needed to advocate for this population when environmental conditions rise.

Almost 2/3 of HRIs were treated and transported by EMS. This speaks to the seriousness of HRI and possible consequences if EMS transport is delayed [10,25]. The need for prehospital health care providers to appropriately recognize HRI, implement treatment quickly, and transport patients to the nearest hospital facility is vital to reduce mortality and morbidity rates [47].

## 5. Conclusions

Heat-related illness affected thousands of individuals each year of the study. All age groups experienced HRI but particularly those 25 to 64 years old. Targeted education to increase public awareness of HRI beyond those traditionally considered vulnerable (children, elderly) may be warranted. Region temperature and urbanicity most likely explain why certain divisions of the US have higher HRI frequency. Yet, all divisions of the US experienced HRI. June through August and 11:00 a.m.–6:59 p.m. are when EMS agencies should be prepared for HRI activations. Heat exhaustion composed the majority of HRI and can adequately be addressed by EMS units with oral or intravenous fluids. Heat stroke, which is deadly, composed the next biggest proportion of HRI cases. All age groups experienced heat stroke. The vast majority of HRIs required treatment and transport, which speaks to the serious nature of HRI. It also may mean those EMS units in high HRI frequency US divisions may need to carry additional treatment interventions for HRI types.

## Figures and Tables

**Table 1:** Heat-related illnesses by race.

	Frequency (*n*)	Percent (%)
American Indian or Alaska Native	196	1.0
Asian	173	0.9
Black or African-American	3889	19.9
Hispanic or Latino	1380	7.1
Native Hawaiian or Other Pacific Islander	66	0.3
White	10,878	55.6
Not Documented	2996	15.3
Total	19,578	100.0

Missing = 15,236.

**Table 2:** Heat-related illnesses by United State census division.

	Frequency (*n*)	Percent (%)
East North Central	3330	9.6
East South Central	2130	6.1
Middle Atlantic	1418	4.1
Mountain	4861	14.0
New England	891	2.6
Pacific	4046	11.6
South Atlantic	11,732	33.7
Territories	16	0.1
West North Central	2598	7.5
West South Central	3792	10.9
Total	34,814	100.0

**Table 3:** Seasonal maximum air temperatures by census division (°C) for 2017–2018 and temperature difference computed as study period minus long-term mean (1981–2010).

DIVISION	Winter	Spring	Summer	Fall	Diff_Win	Diff_Spr	Diff_Sum	Diff_Fall
East North Central	2.1	14.2	27.1	15.6	1.6	−0.2	0.1	−0.2
East South Central	13.5	23.0	31.0	23.0	1.9	0.6	−0.3	0.0
Middle Atlantic	3.1	13.3	26.1	15.8	1.5	−0.5	0.1	0.2
Mountain	5.7	16.9	29.5	17.3	0.7	1.1	1.2	0.5
New England	0.1	10.8	25.0	14.1	1.0	−0.5	0.5	0.5
Pacific	8.6	17.1	29.5	19.2	0.3	0.4	1.6	0.6
South Atlantic	15.4	23.1	30.8	23.9	1.9	0.3	−0.1	0.5
West North Central	1.4	15.3	28.8	15.6	0.8	0.0	0.4	−0.5
West South Central	15.8	25.9	33.8	24.7	1.4	1.4	0.4	−0.2

**Table 4:** Provider impression by age group.

Age Group	Heat Stroke		Heat Syncope		Heat Cramps		Heat Exhaustion		Other	
	*n*	%	*n*	%	*n*	%	*n*	%	*n*	%
<13	530	1.5	14	0.0	16	0.1	1590	4.6	84	0.2
13 to 18	301	0.9	19	0.1	13	0.0	2186	6.3	47	0.1
19 to 24	425	1.2	31	0.1	37	0.1	2455	7.1	53	0.2
25 to 64	2890	8.3	109	0.3	186	0.5	14,987	43.0	317	0.9
>64	1809	5.2	125	0.4	52	0.2	6383	18.3	155	0.4
Total	5955	17.1	298	0.9	304	0.9	27,601	79.3	656	1.9

**Table 5:** Incident disposition frequencies.

	Frequency (*n*)	Percent (%)
Assist	245	0.7
Canceled	75	0.2
Patient Dead at Scene	5	0.0
No Transport *	4244	12.2
Patient Treated, Released	5598	16.1
Patient Treated, Transported	24,539	70.5
Patient Treated, Transported by Private Vehicle	108	0.3
Total	34,814	100.0

* No transport: patient was evaluated but no further treatment needed or refused care.
